# Impact of Storage on the Color of a Green Isotonic Beverage With *β*‐Cyclodextrin/Chlorophyll Complexes

**DOI:** 10.1155/ijfo/6273830

**Published:** 2025-09-04

**Authors:** Claudio Lombardelli, Ilaria Benucci, Chiara Pippolini, Rosita Marabottini, Marco Esti

**Affiliations:** ^1^ Department of Agriculture and Forestry Science (DAFNE), Tuscia University, Viterbo, Italy, unitus.it; ^2^ Department for Innovation in Biological, Agro-Food and Forest Systems (DIBAF), Tuscia University, Viterbo, Italy, unitus.it

**Keywords:** encapsulation, enzyme-assisted extraction, natural colorant, simple mixing, storage stability

## Abstract

This study analyzes the stability of chlorophyll (Chl) from unsold spinach in isotonic beverages, comparing the pigment in its free form with that encapsulated in *β*‐cyclodextrin (*β*‐CD), after determining the inclusion ratio of 1:1. Stability was monitored for 14 days for the crude extract and 30 days for the lyophilized powder under different storage conditions (dark, natural light, and UV light, at 4°C and 25°C). The results prove that both exposure to light, especially UV radiation, and temperature affect the stability of Chl, but encapsulation significantly improves pigment retention. In lyophilized powder form, free Chl is characterized by a slower decline than the wet crude extract, mitigated by the presence of *β*‐CD: After 30 days under the most critical conditions (25°C, UV light), the Chl/*β*‐CD sample retains 30% of the Chl compared to 20% for the free molecule. In this condition, the degradation constant (*k*) of the encapsulated sample is lower than that of the unencapsulated one (0.0366 days^−1^ vs. 0.0612 days^−1^ for the powder and 0.399 days^−1^ vs. 0.644 days^−1^ for the crude extract). The color stability, evaluated using the parameter *Δ* *E*, is also improved by encapsulation, confirming the protective effect of *β*‐CDs.

## 1. Introduction

Isotonic beverages are functional drinks with an osmolarity similar to that of the human body. These beverages are consumed by athletes during physical exercise to prevent dehydration and to replenish essential electrolytes (sodium, potassium, calcium, and magnesium), carbohydrates, and other nutrients depleted through sweating [1]. The appealing organoleptic properties of these beverages are improved by adding sugars, such as glucose and sucrose, but especially synthetic flavors and dyes with sensory characteristics similar to fruit.

However, growing consumer concern about the chemical composition of drinks has led to an increased preference for new formulations based on natural ingredients [2]. As a result, the food industry is focusing on replacing synthetic dyes with natural alternatives. Several studies have tested the efficacy of dyes extracted from natural sources in isotonic drinks, mainly anthocyanins [3].

For example, anthocyanins obtained from ripe fruits of chagalapoli (*Ardisia compressa* K.), in the form of extract and microcapsules, were successfully used by Antonio‐Gómez et al. [4] to color a red isotonic drink.

Other beverages were colored and flavored using Nas Narang (*Citrus madurensis*), a tropical fruit belonging to the Rutaceae family, which is rich in carotenoids and flavonoids responsible for its yellow nuance [5].

The use of chlorophyll (Chl) as natural dyes has not yet been widely studied due to its high instability, as it is susceptible to various external factors such as pH, temperature, and light exposure. This presents a challenge, considering that consumers prefer products with bright and vivid colors [6].

Encapsulation is a technique that offers significant advantages to pigments. Due to the wall materials used, encapsulation can enhance their bioavailability, as well as their thermal and chemical stability, and consequently improve their applicability in the food industry [7].

Various materials are used for coating, with the most common being glaucids, proteins, and lipids. Of particular interest are *β*‐cyclodextrins (*β*‐CDs), which are oligosaccharides obtained by the enzymatic degradation of starch [8].

These molecules have a cyclic structure consisting of seven d‐glucopyranose units linked by *α*‐(1,4)‐glycosidic bonds. They also possess an external hydrophilic surface and an internal cavity with lipophilic properties [9].

The effectiveness of *β*‐CDs in encapsulating natural pigments such as curcumin and anthocyanin has been tested, demonstrating their ability to increase water solubility and improve stability under different storage conditions [10, 11].

To our knowledge, the specific use of Chl, either in free or encapsulated form, for the formulation of a commercial‐type isotonic beverage has not been previously reported, particularly considering the instability of any natural colorants in such matrices. Therefore, the aim of this study was to investigate the use of Chl, enzymatically extracted from spinach and encapsulated in *β*‐CDs as a coloring agent in an isotonic beverage. The results were compared to those obtained using free Chl, evaluating the color stability of the green beverages under different storage conditions (dark/natural light/UV light at different temperature: 4°C, 25°C).

## 2. Materials and Methods

### 2.1. Reagents and Green Extract From Spinach

In this study, the green extract enzymatically recovered from spinach was tested in the form of two different preparations: (i) unrefined crude extract (moisture 85% wet basis) ready to use and (ii) water‐free lyophilized powder (moisture 4.5%).

*β*‐CD (MW 1134.98 g/mol, CAS Number: 7585‐39‐9) was purchased from ACEF S.p.A. (Fiorenzuola d’Arda, Piacenza, Italy). All other reagents, including food‐grade enzymes, were from Merck (Milan, Italy).

### 2.2. Chl Recovery by Enzyme‐Assisted Extraction

The natural green dye (Chl) was recovered from unsold spinach by enzyme‐assisted extraction, following the protocol described by Mazzocchi et al. [12]. Prior to enzymatic treatment, spinach leaves were blanched in boiling water (100°C) for 30 s and then rapidly cooled in ice water. This thermal step was applied to inactivate chlorophyllase and other endogenous degradative enzymes, minimizing pigment breakdown during the extraction process.

The enzymatic extraction was carried out using a McIlvaine buffer (0.1 M, pH 5.5), which provided a stable pH environment known to preserve Chl integrity under mild processing conditions [13]. The enzyme mix, with a total dosage of 30 U/g, consisted of cellulase and pectinase from *Aspergillus niger* and xylanase from *Aspergillus oryzae*. At the end of the extraction, the crude extract (Chl_crude-extract_) was recovered; part of it was used for the preparation of inclusion complexes, while the remainder was lyophilized using a bench‐top freeze dryer (Labconco Corporation, Kansas City, Missouri, United States). The powder obtained (Chl_freeze-dried_) was also used for the preparation of inclusion complexes.

### 2.3. Chl/*β*‐CD Complex in Aqueous Solution

The inclusion complex was prepared by adding 1 mL of Chl stock solution (10^−4^ M in pure ethanol) to 9 mL of *β*‐CD solution at different concentrations (0.5 × 10^−3^, 1.0 × 10^−3^, 0.5 × 10^−2^, 1.0 × 10^−2^, and 1.5 × 10^−2^ M) in water.

The samples and stock solutions were analyzed using a spectrophotometer (UV–visible, Shimadzu UV‐2450, Milan, Italy), with spectra recorded from *λ* 340 to 700 nm using a 1‐mm quartz cuvette (Hellma, Milan, Italy).

The apparent formation constant (K*f*) was determined using the Benesi–Hildebrand method [14] by plotting 1/(*A* − *A*_0_) against 1/[*β* − CD]. A linear correlation was observed, and K*f* was calculated from the slope of the Benesi–Hildebrand plot. The stoichiometric ratio of the inclusion complex between *β*‐CD and Chl was determined using Job’s method: Equimolar solutions of Chl and *β*‐CD (1 × 10^−4^ M) were mixed in varying volume ratios (1:9, 2:8, 3:7, 4:6, 5:5, 6:4, 7:3, 8:2, and 9:1), with a final constant volume of 10 mL. The *β*‐CD solution was then replaced with distilled water, and equal volumes of Chl were added. The absorbance of all solutions was measured in the 340–700 nm range. The stoichiometric ratio was determined by plotting the difference in absorbance of Chl with and without *β*‐CD against the R value (the ratio of Chl to *β*‐CD concentration) [10].

### 2.4. Preparation of the Isotonic Beverage

The isotonic beverage was prepared following a modified version of the recipe described by Antonio‐Gómez et al. [4]: To 100 mL of drinking water, sucrose (7.5 g), sodium chloride (0.020 g), potassium phosphate (0.006 g), potassium sorbate (0.033 g), sodium benzoate (0.015 g), and citric acid (0.04 g) were added to obtain a pH of 3.88.

The isotonic beverage was colored by the addition of an appropriate amount of Chl_crude-extract_ or Chl_freeze-dried_ in free or encapsulated form, in order to obtain the same initial pigment concentration (measured by absorbance Abs).

### 2.5. Encapsulation of Chl in *β*‐CD

The inclusion complex was prepared by simple mixing. Chl_crude-extract_ or Chl_freeze-dried_ was combined with *β*‐CD at a 1:1 molar ratio and manually mixed for 15 min using a mortar and pestle [15].

### 2.6. Stability Study in Isotonic Beverage

The stability of the green‐colored beverage, containing Chl_crude-extract_ or Chl_freeze-dried_ in free form or encapsulated in *β*‐CD, was evaluated under different temperature conditions (4°C and 25°C), in the dark, under UV radiation (9 W UV lamp, with *λ* emission between 300 and 440 nm), and under natural light (LED lamp with *λ* emission between 380 and 800 nm). For the stability trials, aliquots of samples were collected at regular time intervals and subjected to spectrophotometric (Abs spectrum 340 < *λ* < 700 nm) and colorimetric analysis.

### 2.7. UV–Visible Spectrophotometer Assay

Chl content was measured using a UV–Vis spectrophotometer (Abs spectrum 340 < *λ* < 700 nm, Shimadzu, Milan, Italy) and calculated according to the following equation [12]:

(1) Chlμg/g=5.2422.24×Abs664+×Abs649×DF

where Abs_664_ = absorbance at *λ* 664 nm (maximum absorbance of Chl a in ethanol), Abs_649_ = absorbance at *λ* 649 nm (maximum absorbance of Chl b in ethanol), and DF = dilution factor.

### 2.8. Colorimetric Properties

Color analysis was carried out using a CR‐5 colorimeter (Konica Minolta, Tokyo, Japan) based on the CIEL∗a∗b system. The color difference between samples was quantified using the *Δ* *E* parameter, calculated according to the following equation:

(2) ΔE=ΔL∗2+Δa∗2+Δb∗212/.

*L*∗ indicates lightness (0 = black, 100 = white); *a*∗ (−100 to +100) represents the green/red balance (negative = green, positive = red), while *b*∗ (−100 to +100) denotes the blue/yellow balance (negative = blue, positive = yellow).

### 2.9. Chl Degradation Kinetics in Isotonic Beverage

The green color behavior during the storage period was assumed to follow a first‐order kinetic, as previously described for other colorants. The model, based on pigment concentration, is

(3) dC/dt=−kC

where *C* is the residual and initial Chl concentration (micrograms per gram), *t* is the time (days), and *k* is the reaction rate constant (days^−1^).

The half‐life parameter (*t*_1/2_), defined as the time to achieve 50% degradation of pigment, was calculated by the following equation:

(4) t12/=ln 2/k.

### 2.10. Statistical Analysis

Each test was performed in triplicate, and the results were analyzed for statistical significance by using one‐way and two‐way analysis of variance (ANOVA, using Excel extension DSAASTAT) to find the effect of individual factors and their interaction on Chl concentration and stability. Tukey’s post hoc test (HSD) was also performed (*p* < 0.05) using Excel extension DSAASTAT for multiple comparisons between samples.

## 3. Results and Discussion

### 3.1. Chl/*β*‐CD Characterization: Inclusion Complex Formation

Spectrophotometric analysis was used to evaluate the formation of an inclusion complex between Chl recovered by enzyme‐assisted extraction from unsold spinach and *β*‐CD in aqueous solution. The wavelength corresponding to the maximum peak was at 671 nm, differing from the typical one of Chl (*λ* = 654 nm), probably due to the action of the zinc added during the extraction [12, 16–18]. However, it is also reasonable to consider that supramolecular aggregation phenomena may contribute to the observed redshift. In particular, *π*–*π* stacking interactions and the formation of J‐aggregates are known to induce bathochromic shifts in Chl derivatives, especially at high pigment concentration or under specific solvent condition [19, 20]. These self‐assembly processes can alter the electronic environment of the chromophore, potentially leading to spectral features similar to those observed in our system.

The successful formation of the inclusion complex using the same mixing procedure has already been confirmed in a previous study by our group [13], where ^1^H‐NMR spectroscopy revealed characteristic changes in the chemical shift and broadening of *β*‐CD signals, particularly for protons H‐3, H‐5, H‐6, and the anomeric proton H‐1, upon addition of Chl. The most prominent effects were observed in the aliphatic region, suggesting that the phytyl chain of the pigment plays a central role in the host–guest interaction. These results supported the formation of a true inclusion complex rather than a simple physical blend. Additionally, the narrowing of initially broad Chl signals upon titration further supports its encapsulation and increased aqueous solubility.

**Figure 1:**
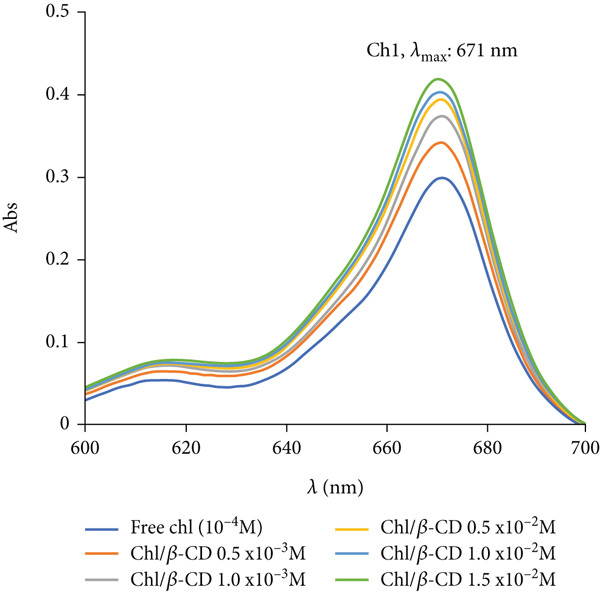
Absorption spectra of Chl stock solution (concentration: 1 × 10^−4^ M) in different *β*‐cyclodextrin (*β*‐CD) amounts: 0.5 × 10^−3^, 1 × 10^−3^, 0.5 × 10^−2^, 1 × 10^−2^, and 1.5 × 10^−2^ M.

In detail, Figure 1 shows the spectra of the Chl stock solution and Chl added to aqueous solutions containing different concentrations of *β*‐CDs (0.5 × 10^−3^, 1.0 × 10^−3^, 0.5 × 10^−2^, 1.0 × 10^−2^, and 1.5 × 10^−2^ M). Even though the maximum of absorption (671 nm) does not change, a hyperchromic shift is observed, which increases as the *β*‐CD concentration increases, confirming the successful formation of the complex.

Determining the inclusion ratio is crucial for optimizing the formation and efficiency of the Chl/*β*‐CD complex. For this purpose, the Benesi–Hildebrand and Job’s methods were used. The first approach demonstrates a linear correlation between 1/(*A* − *A*_0_) and 1/[*β* − CD], with an *R* ^2^ value of 0.99 (Figure 2a) and a formation constant K*f* equal to 1.1 × 10^3^ M^−1^, indicating the production of a 1:1 Chl/*β*‐CD inclusion complex. Consistently, Job’s plot analysis confirms the same stoichiometric ratio, with the highest *R* value at 0.50, validating the 1:1 complex formation (Figure 2b).

Figure 2Stoichiometric ratio of 1:1 between (a) Chl stock solution and *β*‐cyclodextrin (*β*‐CD) and (b) Job’s plot analysis for the complexation Chl/*β*‐CD.

**Figure:**
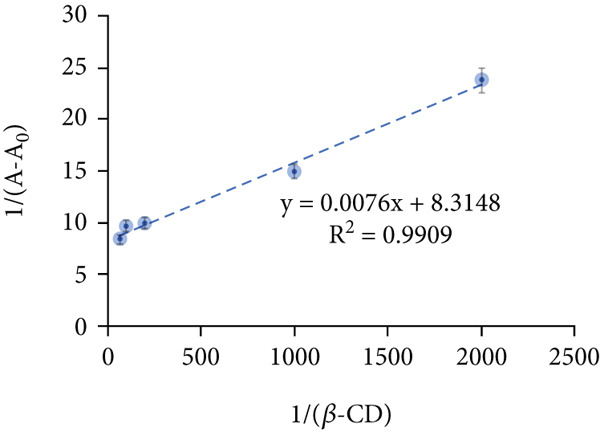
(a)

**Figure:**
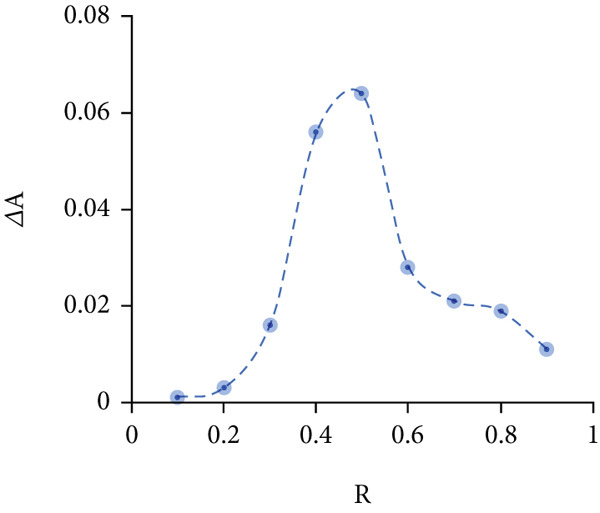
(b)

Conflicting data have been reported in the literature regarding the interaction between Chl and *β*‐CDs. Dentuto et al. [21] found different stoichiometric ratios for Chl inclusion depending on the type of *β*‐CD: a 1:1 complex with heptakis(2,3,6‐tri‐O‐methyl)‐*β*‐CD (TM‐*β*‐CD) and a 1:2 complex with hydroxypropyl‐*β*‐CD (HP‐*β*‐CD), although both have the same cavity size.

The interactions between Chl a and *β*‐CD are primarily hydrophobic, involving the phytyl chain of Chl and the methyl groups inside the cyclodextrin’s cavity. The lower tendency of TM‐*β*‐CD to form hydrogen bonds may explain the 1:1 stoichiometry, a pattern also observed in other porphyrin compounds [22].

### 3.2. Chl Degradation Kinetics in Isotonic Beverage

The instability of Chl in liquid matrices is a limitation for the food industry, because the degradation of this pigment compromises both the visual appearance of the product and the potential beneficial properties [23].

In this study, the green extract enzymatically recovered from spinach has been used to color the isotonic beverage in the form of two different preparations: (i) unrefined crude extract (moisture 85% wet basis) ready to use and (ii) water‐free lyophilized powder (moisture 4.5%).

The stability of Chl in free form and complexed with *β*‐CD was evaluated in an isotonic beverage green colored until the residual Chl content decreased below 50% (14 days for crude extract and 30 days for lyophilized powder).

The Chl_crude-extract_/*β*‐CD and Chl_freeze-dried_/*β*‐CD complexes exhibit a powder recovery of 90.4% and 100%, a loading capacity of 1.4% and 2.5%, an inclusion efficiency of 92% and 96%, and a solubility of 59% and 66%, respectively.

Pigment degradation was monitored under controlled storage conditions (dark, UV light, and natural light at two different temperatures: 4°C and 25°C) using a UV–visible spectrophotometer.

#### 3.2.1. Crude Extract

The variation of Chl content in the isotonic beverage stored in the dark is characterized by a rapid decrease in the first 2 days, slightly greater at 25°C than at 4°C (Figure 3a). The residual pigment concentrations at the end of this initial phase range from 10% (free Chl samples stored at 25°C) to 21% (Chl/*β*‐CD samples stored at 4°C). After this period, Chl degradation follows a more linear trend. Storage at low temperature (4°C), combined with *β*‐CD complexation, proves to be the most effective solution for preserving the pigment. At the end of the experiment, the highest residual Chl content (8.8%) is detected in encapsulated samples stored at 4°C, while under the other conditions, the level of residual free pigment is approximately 2%.

Figure 3Normalized residual concentration (*C*/*C*_0_ *%*) of chlorophyll in free form (free Chl) and in the Chl_crude-extract_/*β*‐CD complex in the isotonic beverage stored at 4°C and 25°C, (a) in the dark, (b) under natural light, or (c) UV light exposure.

**Figure:**
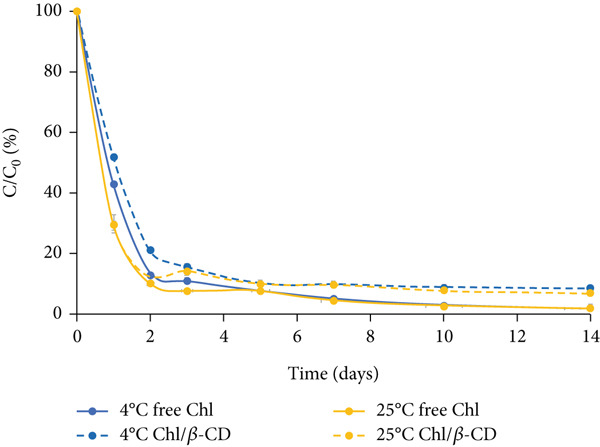
(a)

**Figure:**
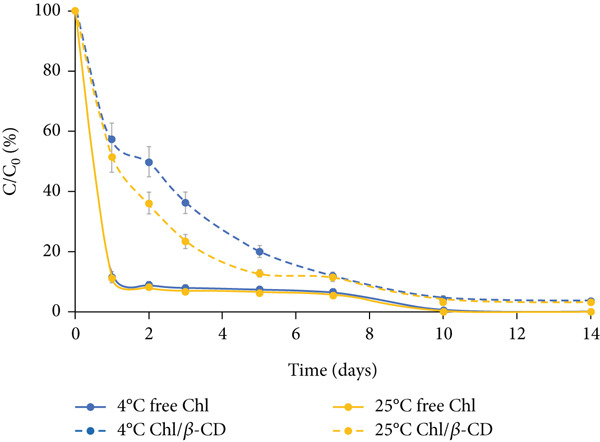
(b)

**Figure:**
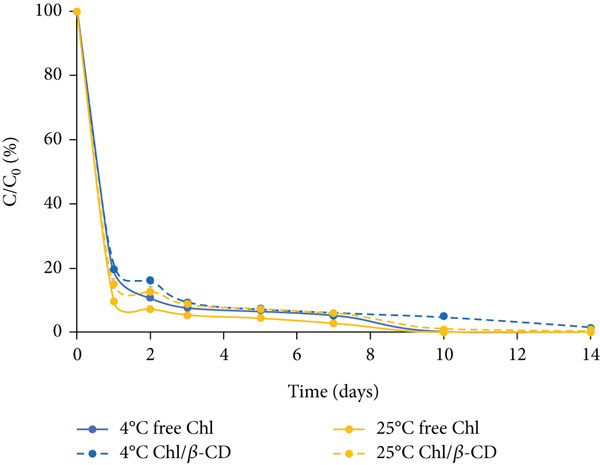
(c)

Under natural light exposure (Figure 3b), Chl degradation is more pronounced compared to the dark storage conditions previously described. Regardless of the storage temperature, free Chl shows a rapid decrease within the first day, with levels reaching approximately 10%. The protective effect of *β*‐CD encapsulation is particularly evident at both temperatures during the first 5 days of storage; however, this protective action gradually decreased. After 2 weeks, free Chl was completely degraded at both 4°C and 25°C, while encapsulated Chl remained detectable, albeit at very low levels (around 3%).

As illustrated in Figure 3c, exposure to UV light represents the most critical condition for pigment stability in the isotonic beverage. Under these conditions, Chl content exhibits a rapid decline from the onset, irrespective of the presence of *β*‐CD. This highlights that UV radiation induces accelerated and aggressive degradation of the pigment and even encapsulation fails to provide a significant protective effect. Importantly, even at 4°C, the encapsulated pigment reaches extremely low levels (approximately 1.5%).

Table 1 presents the values of the degradation rate constant (*k*) and half‐life (*t*_1/2_) calculated for the encapsulated pigment (Chl/*β*‐CD) and free pigment (free Chl) used to color the isotonic drink.

**Table 1:** Degradation kinetic parameters (rate constants [*k*, days^−1^] and half‐life time [*t*_1/2_, days]) of chlorophyll in free form (free Chl) and in the Chl_crude-extract_/*β*‐CD complex in isotonic beverage stored at different temperatures (4°C and 25°C) in the dark, under natural, or UV light. Data were expressed as mean ± standard deviation.

		**T (°C)**	**k** **(days** ^**−1**^ **)**	**t**_1/2_ **(days)**	**R** ^2^
Dark	Free Chl	4	0.347 ± 0.052^a,C^	1.997 ± 0.053^b,B^	0.89
		25	0.364 ± 0.064^a,*γ*^	1.906 ± 0.064^b,*γ*^	0.87
	Chl/*β*‐CD	4	0.248 ± 0.041^b,D^	2.794 ± 0.064^a,A^	0.82
		25	0.266 ± 0.051^b,*δ*^	2.606 ± 0.056^a,*α*^	0.80

Natural light	Free Chl	4	0.534 ± 0.032^b,B^	1.300 ± 0.040^c,C^	0.79
		25	0.564 ± 0.043^a,*β*^	1.229 ± 0.062^c,*δ*^	0.85
	Chl/*β*‐CD	4	0.274 ± 0.042^c,DC^	2.530 ± 0.052^a,AB^	0.98
		25	0.300 ± 0.023^c,*δ*^	2.309 ± 0.031^b,*β*^	0.95

UV light	Free Chl	4	0.583 ± 0.063^a,A^	1.190 ± 0.032^a,C^	0.93
		25	0.644 ± 0.044^a,*α*^	1.076 ± 0.050^d,*δ*^	0.83
	Chl/*β*‐CD	4	0.348 ± 0.022^c,C^	1.990 ± 0.050^a,B^	0.88
		25	0.399 ± 0.061^b,*γ*^	1.739 ± 0.041^b,*γ*^	0.90

*Note:* Statistical significance was determined using ANOVA followed by Tukey’s post hoc test (HSD). Lowercase letters (a–d) indicate significant differences between free Chl and Chl/*β*‐CD across different temperatures. Capital letters (A–C) denote significant differences among samples stored at 4°C, regardless of light exposure (dark, natural light, or UV light). Greek letters (*α*, *β*, *γ*, *δ*, and *ω*) represent significant differences among samples stored at 25°C, also independent of the storage lighting condition (dark, natural light, or UV light) (*p* < 0.05).

The data show that the *k* values for the encapsulated samples are significantly lower than those of the unencapsulated samples (*p* < 0.05), regardless of lighting and temperature conditions. For example, in dark conditions at 4°C, the *k* value is 0.347 days^−1^ for the free pigment and 0.248 days^−1^ for the encapsulated one; in dark conditions at 25°C, the *k* value is 0.364 days^−1^ for the free pigment and 0.266 days^−1^ for the encapsulated one. These results confirm that the presence of *β*‐CD has a significant protective effect on the pigment, slowing down its degradation. To support the kinetic modeling, the coefficient of determination (*R* ^2^) is also reported in Table 1 for each condition. These values describe how well the experimental data fit a first‐order degradation model. The consistently high *R* ^2^ values confirm the appropriateness of this model in describing the degradation behavior of both free and encapsulated Chl.

Furthermore, at the same temperature, both free and encapsulated pigments are more susceptible to degradation when exposed to UV radiation. For the free pigment at 4°C, the *k* value increases progressively from 0.347 days^−1^ in the dark to 0.534 days^−1^ under natural light and up to 0.583 days^−1^ under UV light (*p* < 0.05). A similar trend is observed for the encapsulated pigment, but with lower degradation values: At 4°C, the *k* value is 0.248 days^−1^ in the dark, 0.274 days^−1^ under natural light, and 0.348 days^−1^ under UV light.

#### 3.2.2. Lyophilized Powder

Figure 4 depicts the stability of the green isotonic beverage colored by adding water‐free lyophilized powder, under the same storage conditions (dark, natural light, and UV light at 4°C and 25°C).

Figure 4Normalized residual concentration (*C*/*C*_0_ *%*) of free chlorophyll (free Chl) and Chl in the Chl_freeze-dried_/*β*‐CD complex in the isotonic beverage stored at 4°C and 25°C, (a) in the dark, (b) under natural light, or (c) UV light.

**Figure:**
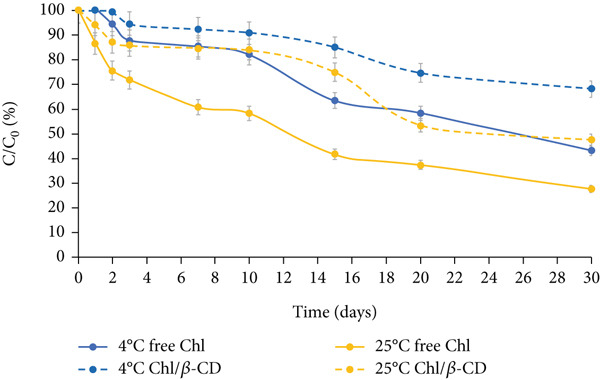
(a)

**Figure:**
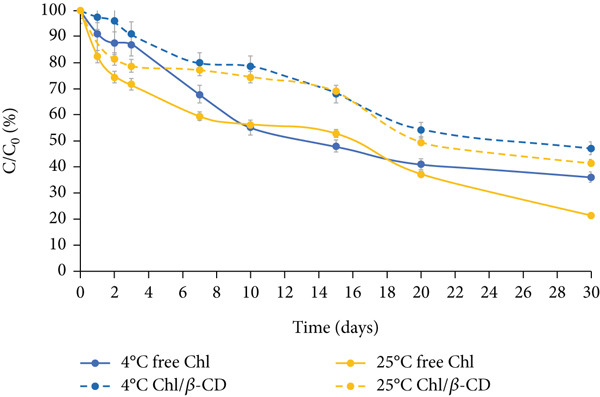
(b)

**Figure:**
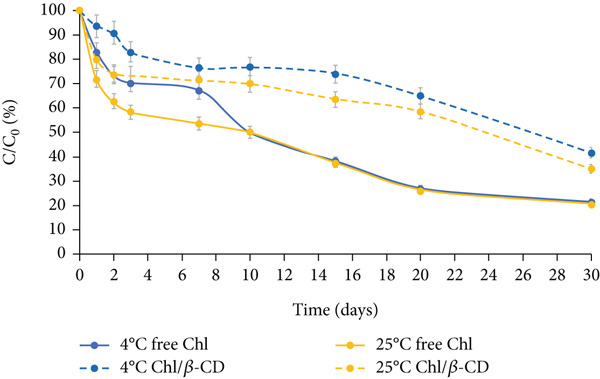
(c)

A much more gradual downward trend is observed compared to the one described for the crude extract, which allowed the storage test to continue for up to 30 days. The enhanced stability observed in the freeze‐dried samples is likely attributable to multiple factors. First, the reduction in water activity resulting from lyophilization significantly limits the molecular mobility and diffusion of degradative agents, thereby slowing down pigment breakdown [24]. In contrast, the higher water content in the crude extract creates favorable conditions for hydrolytic and oxidative reactions. Furthermore, pigment–matrix interactions in the lyophilized powder could also influence pigment stability. During freeze‐drying, components of the spinach matrix (e.g., fibers, proteins, or polyphenols) may form microencapsulation‐like structures or hydrogen‐bonding networks with Chl, which could act as passive protective barriers against light or oxygen [25–27].

Under all tested conditions, the percentage loss of pigment is consistently lower when stored in the dark, compared to natural light or UV light exposure.

After 30 days, free Chl at 4°C shows a decline of 56.6% in the dark (Figure 4a), 63.74% under natural light (Figure 4b), and 78.45% under UV light (Figure 4c), highlighting UV exposure as the most detrimental condition for the pigment stability.

This trend is further confirmed by the *k* values given in Table 2, which suggest the pigment degradation rate constant. The lowest *k* value is observed for storage in the dark (*k* = 0.0274 days^−1^), followed by natural light (*k* = 0.0407 days^−1^), and UV light (*k* = 0.0630 days^−1^) (*p* < 0.05).

**Table 2:** Kinetic degradation parameters (rate constants [*k*, days^−1^] and half‐life time [*t*_1/2_, days]) of free chlorophyll (free Chl) and chlorophyll in the Chl_freeze-dried_/*β*‐CD complex in isotonic beverage stored at 4°C and 25°C, in darkness, under natural, or UV light exposure. Data were expressed as mean ± standard deviation.

		**T (°C)**	**k** **(days** ^**−1**^ **)**	**t**_1/2_ **(days)**	**R** ^2^
Dark	Free Chl	4	0.0274 ± 0.0032^b,C^	25.30 ± 0.03^b,B^	0.99
		25	0.0485 ± 0.0041^a,*β*^	14.29 ± 0.04^c,*Δ*^	0.96
	Chl/*β*‐CD	4	0.0127 ± 0.0041^c,D^	54.58 ± 0.02^a,A^	0.98
		25	0.0254 ± 0.0031^b,*Δ*^	27.29 ± 0.03^b,*α*^	0.96

Natural light	Free Chl	4	0.0407 ± 0.0032^b,B^	17.03 ± 0.03^c,C^	0.96
		25	0.0513 ± 0.0032^a,*β*^	13.51 ± 0.04^d,*Δ*^	0.97
	Chl/*β*‐CD	4	0.0266 ± 0.0022^d,C^	26.06 ± 0.02^a,B^	0.99
		25	0.0308 ± 0.0021^c,*γ*^	22.50 ± 0.03^b,*β*^	0.96

UV light	Free Chl	4	0.0630 ± 0.0032^a,A^	11.00 ± 0.02^c,D^	0.94
		25	0.0612 ± 0.0021^a,*α*^	11.33 ± 0.03^c,*ω*^	0.93
	Chl/*β*‐CD	4	0.0267 ± 0.0041^c,C^	25.96 ± 0.03^a,B^	0.95
		25	0.0366 ± 0.0032^b,*γ*^	20.63 ± 0.04^b,*γ*^	0.92

*Note:* Statistical significance was determined using ANOVA followed by Tukey’s post hoc test (HSD). Lowercase letters (a–d) indicate significant differences between free Chl and Chl/*β*‐CD across different temperatures. Capital letters (A–C) denote significant differences among samples stored at 4°C, regardless of light exposure (dark, natural light, or UV light). Greek letters (*α*, *β*, *γ*, *Δ*, and *ω*) represent significant differences among samples stored at 25°C, also independent of the storage lighting condition (dark, natural light, or UV light) (*p* < 0.05).

After the 1 month of storage period, free Chl at 25°C decreases by 72.3% in the dark, 78.6% under natural light exposure, and 79.5% under UV irradiation. These results highlight not only the susceptibility of free Chl to photoinduced degradation but also its sensitivity to temperature. In fact, the decrease observed at 4°C is consistently lower than that recorded at 25°C, as also reported in the literature [28].

Moreover, in all samples, the highest degradation rates are observed under UV radiation, while the lowest *k* values are recorded in the dark (Table 2, *p* < 0.05). The degradation kinetics reported in Table 2 follow a first‐order model, as confirmed by the high coefficient of determination (*R* ^2^), indicating strong agreement with experimental data.

In the case of lyophilized preparation, the beneficial effect of complexation with *β*‐CD on pigment stability becomes even more evident than that observed for the wet crude extract. By comparing the final concentration of free and encapsulated Chl, the latter always appears more stable (+10%) than the free pigment, also under the most stressful condition (UV light at 25°C) (Figure 4c).

This trend is further confirmed by the lower *k* values calculated for encapsulated pigment, which are approximately halved or nearly halved in comparison to those found for the free form; at 4°C in the dark: 0.0127 days^−1^ versus 0.0274 days^−1^, under natural light: 0.0266 days^−1^ versus 0.0407 days^−1^, and under UV light: 0.0267 days^−1^ versus 0.0630 days^−1^ (Table 2, *p* < 0.05).

Likewise, Agarry et al. [29] used soy protein isolate and chitosan as wall materials to encapsulate Chl, which, compared to its free form, exhibited greater stability during a 21‐day monitoring period in which the pigment was stored at room temperature under intense sunlight.

### 3.3. Stability of the Green‐Colored Isotonic Beverage

Color, being the first perceived element in food and beverages, plays a crucial role in the purchasing decision, creating expectations about the product’s flavor and in the sensory acceptance. If these expectations are not met or the color changes during storage, the consumer may perceive lower quality and choose not to repurchase it [30]. For this reason, it is essential to ensure color stability in isotonic beverages, preserving their original green hue to the greatest extent possible.

*Δ* *E* values for the green isotonic beverage colored by adding the wet crude extract and the lyophilized powder, both in free and encapsulated form, are shown in Tables 3 and 4.

**Table 3:** *Δ* *E* values for crude extract as they are (free Chl) and in the complexes obtained using the crude extract (Chl_crude-extract_/*β*‐CD) in the isotonic beverage stored at 4°C and 25°C, in the dark, under natural, or UV light.

		**Days**							
**Storage conditions**	**Sample**	**0**	**1**	**2**	**3**	**5**	**7**	**10**	**14**
4°C dark	Free Chl		0.0	4.3	7.6	10.4	11.1	13.2	30.6
4°C dark	Chl/*β*‐CD		0.0	3.4	5.1	7.8	10.6	10.6	12.8
25°C dark	Free Chl		2.7	5.0	9.8	11.3	16.4	20.3	20.7
25°C dark	Chl/*β*‐CD		1.3	1.1	6.9	7.0	12.4	16.2	18.0
4°C natural light	Free Chl		10.3	11.5	14.2	23.1	23.2	24.1	24.6
4°C natural light	Chl/*β*‐CD		4.4	3.5	8.5	12.9	16.6	22.5	23.5
25°C natural light	Free Chl		13.0	15.5	20.3	27.2	27.5	26.1	26.5
25°C natural light	Chl/*β*‐CD		10.0	3.9	8.3	15.5	20.2	22.7	23.7
4°C UV light	Free Chl		14.5	17.2	23.0	23.8	25.6	26.6	27.4
4°C UV light	Chl/*β*‐CD		9.5	11.0	18.1	19.1	21.2	19.1	24.4
25°C UV light	Free Chl		23.2	21.9	27.5	23.2	25.8	25.5	28.1
25°C UV light	Chl/*β*‐CD		9.4	18.8	20.1	23.1	23.8	25.3	24.5

**Table 4:** *Δ* *E* values for freeze‐dried samples as they are (free Chl) and in the inclusion complexes (Chl_freeze-dried_/*β*‐CD) stored at 4°C and 25°C, in the dark, under natural, or UV light.

		**Days**								
**Storage conditions**	**Sample**	**0**	**1**	**2**	**3**	**7**	**10**	**15**	**20**	**30**
4°C dark	Free Chl		0.0	2.1	11.3	21.7	24.0	26.3	26.4	27.7
4°C dark	Chl/*β*‐CD		0.0	1.7	6.2	11.4	18.7	19.8	21.1	21.6
25°C dark	Free Chl		5.0	5.8	13.3	13.6	25.9	26.3	27.1	27.9
25°C dark	Chl/*β*‐CD		3.5	5.6	6.3	12.1	18.9	20.2	22.3	22.5
4°C natural light	Free Chl		5.0	8.9	9.6	10.8	18.0	25.1	26.6	34.7
4°C natural light	Chl/*β*‐CD		3.9	6.8	7.6	10.7	13.1	20.0	24.6	22.6
25°C natural light	Free Chl		6.6	9.4	9.8	12.3	18.1	25.4	26.8	36.7
25°C natural light	Chl/*β*‐CD		3.3	7.4	7.9	11.7	12.9	19.7	22.6	24.1
4°C UV light	Free Chl		8.1	9.2	11.8	15.6	19.8	27.9	31.3	37.3
4°C UV light	Chl/*β*‐CD		6.2	6.5	10.4	11.9	13.1	21.9	25.6	25.9
25°C UV light	Free Chl		10.6	11.5	13.7	16.4	21.4	28.8	30.9	38.7
25°C UV light	Chl/*β*‐CD		7.0	10.4	10.5	10.7	15.3	22.8	25.6	26.6

Knowing that higher *Δ* *Ε* values are associated with more evident color changes, previous results are confirmed in both cases: Exposure to natural light and, especially, to UV radiation led to the most pronounced color changes, confirming that light exposure, particularly UV light, is one of the main destabilizing factors for Chl.

Under all tested conditions, free Chl exhibited higher *Δ* *E* values compared to encapsulated Chl, demonstrating that *β*‐CD encapsulation may partially mitigate color variation over time. However, the protective efficacy of *β*‐CD is strongly influenced by storage conditions, particularly temperature and light exposure.

**Figure 5:**
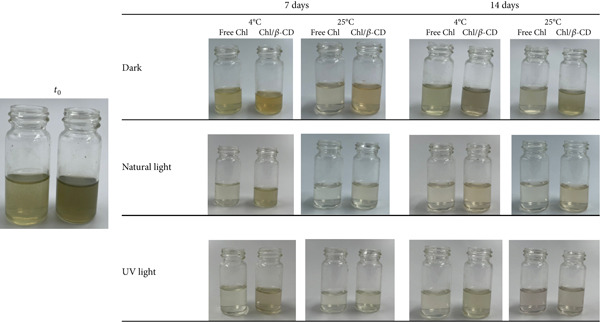
Isotonic beverage tinted with Chl_crude-extract_ or Chl_crude-extract_/*β*‐CD following 7‐ and 14‐day storage at 4°C and 25°C, in the dark, under either natural, or UV light conditions.

Figure 5 shows the chromatic evolution and visual stability of the isotonic drink, colored with Chl from crude extract, evaluated both in the middle and at the end of the monitoring period. Samples stored under light exposure, including both natural light and UV radiation, exhibit noticeable color changes toward yellow‐brown hues. This phenomenon is attributed to the photodegradation of Chl, which undergoes a progressive conversion into pheophytin derivatives, responsible for this coloration [18].

Temperature is also a critical factor in pigment destabilization. Specifically, the combination of room temperature (25°C) and light exposure, particularly UV radiation, represents the most critical condition for color stability. As depicted in the images, samples subjected to these conditions undergo a near‐complete loss of their original color (Figure 5).

**Figure 6:**
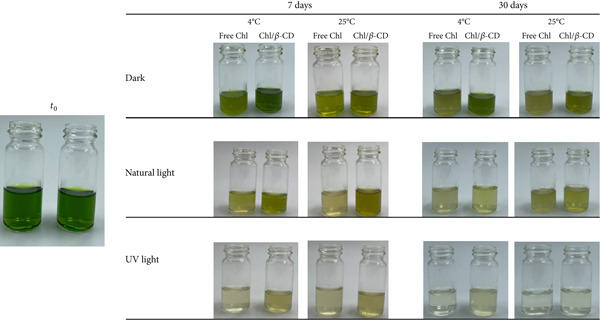
Isotonic beverage colored by adding Chl_freeze-dried_ or Chl_freeze-dried_/*β*‐CD after 7‐ and 30‐day storage at 4°C and 25°C, in the dark, under natural, or UV light.

However, Figure 6 shows the chromatic evolution of isotonic beverages colored with Chl in powder, after 7 days and at the end of the monitoring period.

Comparing these samples with those containing Chl in crude extract (Figure 5), several differences may be observed. For instance, the initial color appears different: In the samples colored by adding the free crude extract, the nuance is more opaque and less bright, whereas in the samples containing lyophilized Chl, it is more green, intense, and vibrant.

In the isotonic beverage with lyophilized Chl, the color change occurs more gradually. After 7 days, the samples still retain a relatively strong color, although brownish shades are already visible, especially in the presence of free pigment under the most critical conditions (25°C and UV light exposure). In all cases, the beneficial effect of *β*‐CD is consistently evident, as it effectively reduces the rate and intensity of the color change process.

Alongside the presence of *β*‐CD, dark storage also provides a significant advantage: Even after 30 days, samples still retain an appreciable green color.

## 4. Conclusions

This study highlights the crucial role of *β*‐CD encapsulation in enhancing the stability of Chl in isotonic beverages. The beverages, colored with Chl_crude-extract_ and Chl_freeze-dried_ in both free and encapsulated forms, were stored under various conditions: darkness, natural light, and UV light, at two different temperatures (4°C and 25°C).

The results clearly demonstrate that exposure to light, especially UV radiation, accelerates Chl degradation, resulting in noticeable color changes, primarily toward yellow‐brown hues. Temperature also plays a significant role in pigment destabilization, with degradation occurring more rapidly at 25°C compared to 4°C.

When used to color the isotonic beverage, lyophilized Chl powder exhibited a more gradual decline in pigment content over time compared to the crude extract. Additionally, the lyophilized form provided a more intense and vibrant green color at the start of the experiment, which was better preserved during storage.

Encapsulation with *β*‐CD showed a remarkable protective effect, slowing the degradation process and minimizing the rate of color change. Encapsulated Chl, in both crude extract and lyophilized powder forms, exhibited lower degradation rates compared to free Chl.

In conclusion, *β*‐CD encapsulation significantly enhances the stability of Chl in isotonic beverages by creating a protective barrier against degradation induced by light and temperature, both key factors affecting color stability. The combination of *β*‐CD encapsulation and low‐temperature storage proves to be the most effective strategy for preserving the pigment and maintaining the visual attributes of green beverages.

## Ethics Statement

Ethical approval was not required for this study.

## Conflicts of Interest

The authors declare no conflicts of interest.

## Author Contributions

**Ilaria Benucci:** conceptualization, validation, data curation, writing—review and editing, visualization, supervision, funding acquisition; **Marco Esti:** conceptualization, validation, resources, writing—review and editing, supervision, project administration, funding acquisition; **Claudio Lombardelli:** methodology, investigation, data curation, writing—original draft preparation, visualization; **Chiara Pippolini:** methodology, formal analysis, investigation, data curation, writing—original draft preparation, visualization; **Rosita Marabottini:** methodology, formal analysis

## Funding

This work was supported by Project ECS 0000024 Rome Technopole (CUP B83C22002820006), funded by the PNRR Mission 4 Component 2 Investment 1.5 from the European Union—NextGenerationEU, with additional support from Unicoop Tirreno SC (Viterbo, Lazio region, Italy).

## Data Availability

The data that support the findings of this study are available from the corresponding author upon reasonable request.

## References

[1] Orrù S. , Imperlini E. , Nigro E. , Alfieri A. , Cevenini A. , Polito R. , Daniele A. , Buono P. , and Mancini A. , Role of Functional Beverages on Sport Performance and Recovery, Nutrients. (2018) 10, no. 10, 10.3390/nu10101470, 2-s2.0-85054715653, 30308976. PMC6213308 30308976

[2] Cui P. , Li M. , Yu M. , Liu Y. , Ding Y. , Liu W. , and Liu J. , Advances in Sports Food: Sports Nutrition, Food Manufacture, Opportunities and Challenges, Food Research International. (2022) 157, 111258, 10.1016/j.foodres.2022.111258, 35761570. 35761570

[3] Wallace T. C. and Giusti M. M. , Selective Removal of the Violet Color Produced by Anthocyanins in Procyanidin-Rich Unfermented Cocoa Extracts, Journal of Food Science. (2011) 76, no. 7, C1010–C1017, 10.1111/j.1750-3841.2011.02322.x, 2-s2.0-80053091568, 22417537. 22417537

[4] Antonio-Gómez M. V. , Salinas-Moreno Y. , Hernández-Rosas F. , Herrera-Corredor J. A. , and Contreras-Oliva A. , Color and Stability of Anthocyanins of Chagalapoli (*Ardisia compressa* K.) Fruit Added to an Isotonic Beverage as Microcapsules and as Free Extract, Food. (2023) 12, no. 10, 10.3390/foods12102009. PMC10217480 37238826

[5] Idangodage I. P. A. , de Siva A. B. C. G. J. , Herath H. M. T. , and Jayasinghe J. M. J. K. , Development and Physico-Chemical Evaluation of an Isotonic Nas Narang (*Citrus madurensis*) Sports Drink, Journal of Advances in Food Science & Technology. (2023) 10, no. 2, 75–85, 10.56557/jafsat/2023/v10i28130.

[6] Harsito C. , Prabowo A. R. , Prasetyo S. D. , and Arifin A. , Enhancement Stability and Color Fastness of Natural Dye: A Review, Open Engineering. (2021) 11, no. 1, 548–555, 10.1515/eng-2021-0055.

[7] Ghosh S. , Sarkar T. , Das A. , and Chakraborty R. , Natural Colorants From Plant Pigments and Their Encapsulation: An Emerging Window for the Food Industry, LWT. (2022) 153, 112527, 10.1016/j.lwt.2021.112527.

[8] Maw P. D. and Jansook P. , Cyclodextrin-Based Pickering Nanoemulsions Containing Amphotericin B: Part I. Evaluation of Oil/Cyclodextrin and Amphotericin B/Cyclodextrin Inclusion Complexes, Journal of Drug Delivery Science and Technology. (2022) 68, 103118, 10.1016/j.jddst.2022.103118.

[9] Dhiman P. and Bhatia M. , Pharmaceutical Applications of Cyclodextrins and Their Derivatives, Journal of Inclusion Phenomena and Macrocyclic Chemistry. (2020) 98, no. 3-4, 171–186, 10.1007/s10847-020-01029-3.

[10] Benucci I. , Mazzocchi C. , Lombardelli C. , del Franco F. , Cerreti M. , and Esti M. , Inclusion of Curcumin in b-Cyclodextrin: A Promising Prospective as Food Ingredient, Food Additives & Contaminants: Part A. (2022) 39, no. 12, 1942–1952, 10.1080/19440049.2022.2135764, 36255357. 36255357

[11] Fernandes A. , Ivanova G. , Brás N. F. , Mateus N. , Ramos M. J. , Rangel M. , and de Freitas V. , Structural Characterization of Inclusion Complexes Between Cyanidin-3-*O*-Glucoside and *β*-Cyclodextrin, Carbohydrate Polymers. (2014) 102, 269–277, 10.1016/j.carbpol.2013.11.037, 2-s2.0-84890954600, 24507282. 24507282

[12] Mazzocchi C. , Benucci I. , Lombardelli C. , and Esti M. , Enzyme-Assisted Extraction for the Recovery of Food-Grade Chlorophyll-Based Green Colorant, Food. (2023) 12, no. 18, 10.3390/foods12183440, 37761155. PMC10529526 37761155

[13] Lombardelli C. , Benucci I. , Pippolini C. , and Esti M. , Guest–Host Inclusion Complexes of *β*-Cyclodextrin With Commercial Oil–Soluble Copper Chlorophyll and Natural Chlorophyll Recovered by Enzyme-Assisted Extraction, Food Frontiers. (2025) 10.1002/fft2.70077.

[14] Marcolino V. A. , Zanin G. M. , Durrant L. R. , Benassi M. D. T. , and Matioli G. , Interaction of Curcumin and Bixin With *β*-Cyclodextrin: Complexation Methods, Stability, and Applications in Food, Journal of Agricultural and Food Chemistry. (2011) 59, no. 7, 3348–3357, 10.1021/jf104223k, 2-s2.0-79953836681, 21381747. 21381747

[15] Tao F. , Hill L. E. , Peng Y. , and Gomes C. L. , Synthesis and Characterization of *β*-Cyclodextrin Inclusion Complexes of Thymol and Thyme Oil for Antimicrobial Delivery Applications, LWT-Food Science and Technology. (2014) 59, no. 1, 247–255, 10.1016/j.lwt.2014.05.037, 2-s2.0-84903790530.

[16] Fathurrohmah N. N. , Murti S. T. C. , and Suryani C. L. , Effect of Zn–Chlorophyll Complexes Formation on the Color Stability of Pandan (*Pandanus amaryllifolius*) Leaf Extract, Food Science and Technology. (2023) 11, no. 3, 161–167, 10.13189/fst.2023.110304.

[17] Lombardelli C. , Mazzocchi C. , Benucci I. , and Esti M. , Stabilized Chlorophyll-Based Food Colorants From Spinach: Kinetics of a Tailored Enzymatic Extraction, Journal of Food Science. (2024) 89, no. 9, 5270–5279, 10.1111/1750-3841.17269, 39086064. 39086064

[18] Mazzocchi C. , Møller A. H. , Benucci I. , Esti M. , and Dalsgaard T. K. , Enhancing the Water Solubility and Stability of Zinc Modulated Chlorophyll by Alginate-Whey Protein Isolate Complexes at Different pH Values, Food Hydrocolloids. (2025) 163, 111115, 10.1016/j.foodhyd.2025.111115.

[19] Hisahara Y. , Nakano T. , and Tamiaki H. , J-Aggregation of Chlorophyll-a Derivatives Inserting Fluorinated Phenylene Linker, Chemistry Letters. (2025) 54, no. 2, 10.1093/chemle/upaf023.

[20] Silva P. J. , Osswald-Claro M. , and Castro Mendonça R. , How to Tune the Absorption Spectrum of Chlorophylls to Enable Better Use of the Available Solar Spectrum, PeerJ Physical Chemistry. (2022) 4, no. 26, 10.7717/peerj-pchem.26.

[21] Dentuto P. L. , Catucci L. , Cosma P. , Fini P. , Agostiano A. , D’Accolti L. , Trevithick-Sutton C. C. , and Foote C. S. , Effect of Cyclodextrins on the Physicochemical Properties of Chlorophyll a in Aqueous Solution, The Journal of Physical Chemistry B. (2005) 109, no. 3, 1313–1317, 10.1021/jp047132p, 2-s2.0-13444274676, 16851096. 16851096

[22] Oliveri V. , Puglisi A. , and Vecchio G. , New Conjugates of *β*-Cyclodextrin With Manganese (III) Salophen and Porphyrin Complexes as Antioxidant Systems, Dalton Transactions. (2011) 40, no. 12, 2913–2919, 10.1039/C0DT01480J, 2-s2.0-79952651025, 21321734. 21321734

[23] Ferruzzi M. and Blakeslee J. , Digestion, Absorption, and Cancer Preventative Activity of Dietary Chlorophyll Derivatives, Nutrition Research. (2007) 27, no. 1, 1–12, 10.1016/j.nutres.2006.12.003, 2-s2.0-33846576700.

[24] Zäh M. , Brandenbusch C. , Groël S. , Winter G. , and Sadowski G. , Water Activity as an Indicator for Antibody Storage Stability in Lyophilized Formulations, Molecular Pharmaceutics. (2025) 22, no. 2, 918–926, 10.1021/acs.molpharmaceut.4c01106. 39809457 PMC11795528

[25] Senge M. O. , Ryan A. A. , Letchford K. A. , MacGowan S. A. , and Mielke T. , Chlorophylls, Symmetry, Chirality, and Photosynthesis, Symmetry. (2014) 6, no. 3, 781–843, 10.3390/sym6030781, 2-s2.0-84907710697.

[26] Terakita A. , Matsunaga H. , and Handa T. , The Influence of Water on the Stability of Lyophilized Formulations With Inositol and Mannitol as Excipients, Chemical and Pharmaceutical Bulletin. (2009) 57, no. 5, 459–463, 10.1248/cpb.57.459, 2-s2.0-65949095634, 19420775. 19420775

[27] Östbring K. , Sjöholm I. , Rayner M. , and Erlanson-Albertsson C. , Effects of Storage Conditions on Degradation of Chlorophyll and Emulsifying Capacity of Thylakoid Powders Produced by Different Drying Methods, Food. (2020) 9, no. 5, 10.3390/foods9050669, 32455958. PMC7278877 32455958

[28] Kwartiningsih E. , Ramadhani A. N. , Putri N. G. A. , and Damara V. C. J. , Chlorophyll Extraction Methods Review and Chlorophyll Stability of Katuk Leaves (*Sauropus androgynous*), Journal of Physics: Conference Series. (2021) 1858, no. 1, 012015, 10.1088/1742-6596/1858/1/012015.

[29] Agarry I. E. , Wang Z. , Cai T. , Kan J. , and Chen K. , Chlorophyll Encapsulation by Complex Coacervation and Vibration Nozzle Technology: Characterization and Stability Study, Innovative Food Science & Emerging Technologies. (2022) 78, 103017, 10.1016/j.ifset.2022.103017.

[30] Stich E. , Food Color and Coloring Food: Quality, Differentiation and Regulatory Requirements in the European Union and the United States, Handbook on Natural Pigments in Food and Beverages, 2016, Woodhead Publishing, 3–27, 10.1016/B978-0-08-100371-8.00001-4, 2-s2.0-84979946026.

